# Psychological Aspects, Physical Activity Levels and Overweight Concerns: A Cross-over Study of Brazilian Adolescents

**DOI:** 10.2174/17450179-v18-e221020-2022-15

**Published:** 2022-10-18

**Authors:** Kamila Souza Santana, Sidnei Jorge Fonseca Junior, Cássia Queiroz, Aldair José de Oliveira, Sergio Machado, Geraldo de Albuquerque Maranhão Neto

**Affiliations:** 1 Salgado de Oliveira University, Niterói, Brazil; 2 Rio de Janeiro State University, Rio de Janeiro, Brazil; 3 Graduate Program of Physical Activity Sciences, Salgado de Oliveira University, Niterói, Brazil; 4 Rural Federal University of Rio de Janeiro, Rio de Janeiro, Brazil; 5 Department of Sports Methods and Techniques, Federal University of Santa Maria, Santa Maria, Brazil; 6 Laboratory of Physical Activity Neuroscience, Neurodiversity Institute, Queimados- RJ , Brazil; 7 Kardiovize Study, International Clinical Research Centre, St. Anne's University Hospital, Brno, Czech Republic

**Keywords:** Anxiety, Depression, Overweight, Physical activity, Body weight, Adolescents

## Abstract

**Introduction:**

This study aims to identify levels of anxiety, depression, body weight, and levels of physical activity, as well as to verify possible associations among these variables in Brazilian adolescents. It is a cross-sectional study with a convenience sample of 291 Brazilian adolescents aged 13 to 15 years old (13.75±0.80 years old).

**Methods:**

Researchers assessed students by filling out questionnaires of anxiety and depression symptoms, as well as the level of physical activity and checking anthropometric measures. Parametric and non-parametric statistics were used to compare groups divided by psychological, physical and overweight factors for boys and girls, adopting a significance level of 95%.

**Results:**

Results showed higher waist circumference and physical activity levels for boys (p<0.01) and higher scores for anxiety and depressive symptoms questionnaires for girls (p<0.01). In addition, a low prevalence of depression was observed in boys (0.69%/CI0.03-4.36). For anxiety and depression variables in males and females with and without overweight and physically active and inactive, there were significant differences between groups (p < 0.05), but not within groups.

**Conclusion:**

The identification of a higher prevalence of girls with anxiety and depression was observed in this study and reinforced this information already demonstrated in the scientific literature. In general, the prevalence of depression was low in boys, while the prevalence of overweight, sedentary lifestyle, anxiety and depression were higher in girls.

## 1 INTRODUCTION

Mental health and its relations with overweight is a relevant and contemporary discussion regarding children and adolescents [1-3]. Thus, expanding the understanding of overweight and obesity and the most common mental disorders, *e.g*. depression and anxiety, allows researchers to deeply advance into this issue. Due to overweight and obese children and adolescents end up exposed to several psychological and behavioral problems (*e.g*., depression, reduction of self-esteem and problems associated with body image), which interfere with their interpersonal relationships and school performance [4]. In particular, adolescence is a crucial period of life, where many changes occur, making mental health a determining factor in this age range for later life.

A possible strategy against overweight and obesity is to encourage children and adolescents to practice physical activities. It is known that the promotion of a healthier lifestyle helps to control body weight, extremely important not only for physical health [5], but also for mental health [6-8]. However, approximately 81% of adolescents between 13 and 15 years do not follow the recommendations of the World Health Organization (WHO) and the American College of Sports Medicine (ACSM) for physical activity practice, regardless of socioeconomic level [9-11].

High levels of physical activity have a positive association with mental health [9, 12], that is, evidence proposes that the practice of physical activity increases cognition and mental health through changes in brain functioning [13-17]. The opposite is also true, low levels of physical activity practice were associated with lower psychosocial well-being levels [18] and depression [19]. However, these associations have not been extensively studied [20] and need to be better investigated in this population, due to the high prevalence of childhood obesity around the world [21, 22]. Within this context, our study aims to determine the association between physical activity levels, body weight, depressive and anxiety symptoms among Brazilian adolescents.

## 2 MATERIALS AND METHODS

### 2.1 Sample

In this cross-sectional study, a convenience sample consisted of 291 adolescents aged 13 to 15 years old (13.75±0.80 years old), enrolled in 9 public schools from Niterói city, located in the state of Rio de Janeiro, who agreed to carry out the study. Inclusion criteria adopted were: being enrolled in the public school system from Niterói city, having authorization from the school's pedagogical team, being of the established age, and complying with all steps of data collection, including the delivery of the Informed Consent Form and Clarified signed. Students with any disability that prevented completing the questionnaires or contained the anthropometric diagnosis were excluded.

### 2.2 Experimental Procedures

The project was submitted and approved by the Human Research Ethics Committee of Salgado de Oliveira University. Once approved, it was sent to the Municipal Education Foundation (FME) and authorization for data collection was generated. The responsible for students selected to participate in this research was informed by the directions of each school about data collection. After all researchers responsible for data collection had undergone prior training, they applied to adolescents the questionnaires Multidimensional Anxiety Scale for Children (MASC), Children’s Depression Inventory (CDI) and Physical Activity Questionnaire for Adolescents (PAQA), and collected anthropometric measurements of weight, height, and waist circumference in only one session.

### 2.3 Instruments

#### 2.3.1 Multidimensional Anxiety Scale for Children (MASC)

To assess the level of anxiety, the Multidimensional Anxiety Scale for Children (MASC) was used, validated for the Brazilian child population by Nunes [23]. MASC is a self-answer questionnaire with four main factors: physical symptoms, avoidance of danger, social anxiety and separation anxiety. Among these four factors, 'physical symptoms', 'danger avoidance' and 'social anxiety' are subdivided into 2 more factors, making a total of 39 items [24]. These items are answered by children/adolescents who can answer the affirmative, selecting alternatives from 0 to 3. A score of 56 was established as a cutoff point and adolescents with higher results were considered to be anxious [24].

#### 2.3.2 Children’s Depression Inventory (CDI)

For this assessment, the Children's Depression Inventory (CDI) consisted of 20 questions, with depressive symptoms marked using a Likert scale from 0 to 2 points. In the CDI, in relation to symptoms, 0 represents absence, “1” presence and “2” the greatest severity. The respondent should mark the alternative that best describes their feelings regarding the past two weeks. Its items address affective reactions, cognitive, behavioral and somatic aspects. The cutoff point adopted was 17 points and the adolescents who obtained the highest score were considered individuals with a higher index of symptoms of depression [25].

#### 2.3.3 Physical Activity Questionnaire for Adolescents (PAQ-A)

To collect information on the level of physical activity, the Physical Activity Questionnaire for Adolescents (PAQ-A) was used, a translation and cultural adaptation of the Self-Administered Physical Activity Checklist [26]. The PAQ-A consists of a list of physical activities most practiced by Brazilian adolescents (culturally adapted), taking into account the age group and context of the “target” population, which measures the time, frequency and duration of the activity. This instrument allows you to estimate and classify the level of physical activity according to health recommendations. Furthermore, it has acceptable levels of reliability and validity in both younger (10-14 years) and older (14-19 years) adolescents. Adolescents with physical activity equal to or greater than 300min/week and others as insufficiently active were considered sufficiently active [27].

### 2.4 Anthropometry and Overweight Assessment

Body weight was measured using an OMRON^®^ brand scale with a precision level of 0.1 kg. Adolescents were weighed with as little clothing as possible, barefoot, with no objects in their hands and pockets. Body height was measured using an improvised stadiometer with a Sanny Medical Starrett^®^ (SMS^®^) anthropometric tape measure with 0.1cm precision, which was affixed to the wall for measurements. Each individual was positioned in the Frankfurt Plan, hands along the body, feet together and centered, with buttocks and heels touching the wall [28].

The body mass index (BMI) was calculated by the ratio of body mass and height squared, using the 85th percentile cutoff point, according to the (WHO) [26]. Thus, the following values ​​were defined as overweight: 13 years old, boys, from 21.93 kg/m^2^; girls from 23.08 kg/m^2^; 14 years old, boys, from 22.77 kg/m^2^; girls from 23.88 kg/m^2^; 15 years old, boys, from 23.63 kg/m^2^; girls from 24.29 kg/m^2^.

Waist circumference (WC) measurement was obtained with an anthropometric tape with the precision of 0.1cm (SMS^®^) being placed horizontally, at the midpoint between the lower edge of the last rib and the iliac crest, with the adolescent standing in foot and arms relaxed along the body. The following cutoff points were used to classify excess central adiposity: boys, from 76.8 cm and girls, from 75.6 cm, among those aged 13 years; boys, from 79 cm and girls, from 77 cm, among those who were 14 years old; boys, from 81.1 cm and girls, from 78.3, among those aged 15 [29].

### 2.5 Statistical Analysis

The mean and standard deviation were used to describe the parametric variables. Median and interquartile differences were used for non-parametric data or discrete variables. The prevalence of overweight, physical inactivity, anxiety and depression were described using the respective confidence interval. Statistical comparisons between boys and girls using parametric (Student's t-test) and non-parametric (Mann-Whitney test) procedures were also used. Between groups divided by sex and presence of excess weight and physical activity level, comparisons of levels of anxiety and depression were performed using Boxplot charts and the Kruskal-Wallis test, with the comparison of pairs with values ​​adjusted for the number of comparisons being used to identify between which groups the differences occurred. The multiple covariance analysis (MANCOVA) considering the presence or not of mental disorders (anxiety and depression) as independent variables, excess weight and activity practice time as dependent variables and sex as a covariate was also used. In all statistical comparisons, P value < 0.05 was considered for statistical significance. SPSS version 20 for Windows and R version 3.5.2 were used for data analysis.

## 3 RESULTS

Table **1** shows the descriptive results for boys and girls and the general results for variables related to anthropometry, anxiety, depression and level of physical activity. Mean and standard deviation were used for parametric variables. Median and interquartile differences were used for non-parametric data or discrete variables. Statistical comparisons between boys and girls using parametric and non-parametric procedures were also carried out, demonstrating greater height, waist circumference, level of physical activity and metabolic equivalent (METs) for boys (p<0.01) and higher scores were obtained in the anxiety and depression questionnaires for girls (p<0.01).

The prevalence values, accompanied by their respective confidence intervals, of the variables overweight, abdominal obesity, physical inactivity, anxiety and depression can be observed in Table **2**. It is important to note that variables associated with obesity, such as overweight and abdominal obesity showed different characteristics between boys and girls, although the confidence interval was high. Girls had a higher prevalence of physical inactivity, anxiety and depression. In addition, the low value of depression observed in boys should be highlighted.

In general, in Figs. (**1**-**3**), significant differences (p<0.05) were observed between at least two groups. Descriptive statistics, through boxplots, also allow for the visualization of outliers and favor the interpretation of data, which, strengthened by the results in Table **1**, suggest differences between boys and girls in the variables anxiety and depression. Fig. (**1**) shows the results of anxiety and depression for boys and girls with and without excess weight. Significant differences can be observed between at least two groups compared both in the assessment of anxiety [X^2^(3)=21.752; p<0.01] and in depression [X^2^(3)=13.218; p<0.01]. The comparison of pairs with values ​​adjusted for the number of comparisons, in the anxiety variable, showed significant differences between the group of boys without excess weight and the groups of girls, both without (p=0.001) and with (p=0.009) overweight; in the variable depression, considering the score based on symptoms, significant differences occurred between the groups of boys and girls without excess weight (p=0.009).

The results shown in Fig. (**2**) represent the assessment of anxiety and depression with groups divided into girls and boys, with and without the presence of excess weight, represented by abdominal obesity. Significant differences can be observed between groups both in the assessment of anxiety [X^2^(3)=21.225; p<0.01] and in depression [X^2^(3)=13.928; p<0.01]. In the anxiety variable, the comparison of pairs with values adjusted for the number of comparisons, showed significant differences between the group of boys without abdominal obesity and the groups of girls, both those without (p=0.001) and those with (p=0.021) obesity abdominal; in the variable depression, significant differences occurred between the groups of boys and girls without abdominal obesity (p=0.002).

In Fig. (**3**), the comparisons of girls and boys divided by the level of physical activity that divided the sample into physically active and inactive, also showed significant differences in the anxiety variables [X^2^(3)=21.370; p<0.01] and depressive symptoms [X^2^(3)=14,811; p<0.01]. In the variable anxiety, the adjusted pair comparisons showed significant differences between the inactive male group and the inactive (p = 0.001) and physically active (p = 0.001) female groups; the group of active boys showed significant differences only with girls also physically active (p = 0.042). In the variable depression, both the group of active (p = 0.011) and inactive (p = 0.005) boys showed significant differences in the score of depressive symptoms with the group of physically inactive girls.

MANCOVA, with the sample divided into adolescents without anxiety and/or depression (*e.g*. common mental disorders), with anxiety and/or depression, was used to do comparisons in overweight variables and minutes practicing physical activity, with the covariate sex for control, demonstrating influence in the compared variables (p < 0.001). After observing that the WC and BMI variables could not be treated together as dependent by presenting multicolinearity, the results showed that there is no influence of anxiety or depression (p > 0.05) in the variables WC and minutes practicing physical activity [Pillai trace = 0.016; F (4,572) = 1.555], as well as in the BMI variables and minutes practicing physical activity [Pillai trace = 0.015; F (4,572) = 1.555] (Table **3**).

## 4 DISCUSSION

The results of this study present relevant information about adolescents from Brazilian public schools, favoring a better understanding of the relationship between psychological aspects, overweight and levels of physical activity in this age group. Although the literature presents the possibility that excess weight and physical activity level are associated with anxiety and depression [30-33], the results within the same sex did not show significant differences, with significant differences being found only in comparisons between groups of girls and boys. With the sample divided into adolescents with and without these common mental disorders and with control of the sex variable, no differences were observed in the variables of physical activity practice and exception of weight. Studies carried out with Brazilian adolescents show that females are more likely to be more anxious, depressed and more physically inactive than males [3, 8]. However, the prevalence of obesity has increased in males according to cross-sectional and temporal trends studies, with recent results showing balanced values ​​or with small differences in relation to females [34, 35].

Higher prevalence of anxiety and depression are common in females [3, 36]. A population-based study with a stratified sample of Brazilian schoolchildren corroborates the results of this study, presenting data from different regions of Brazil and identifying the prevalence of mental disorders of 26.7% between 12 and 14 years old and 33.6% between 15 and 17 years of age, being always higher in females [3]. When reflecting on anxiety and depression together, the higher prevalence of these disorders found in girls may be due to biological issues such as hormonal changes during adolescence that lead to changes in mood, neuroendocrine differences and differences in reproductive structures and functions that can also be responsible for such results [37, 38]. The literature also reports the effects of psychological and social variables that include the low social status of girls, in addition to rules and upbringing differentiated between the sexes, greater absorption of stressful events and the concern and demand for girls' body image [38, 39].

Another important observation in this study was the prevalence of depression close to zero in children, in addition to the fact that even with a low value, it should not be neglected. Although the results of the prevalence of depression may vary according to the characteristics of the population studied, any increase in cases in boys and girls should be considered of concern due to the additional dangers that depression can elicit, such as suicide ideation and increased consumption of licit and illicit drugs [1,2,40]. However, when it comes to anxiety, the result found must be considered relevant and deserves special attention for girls and boys. Studies in different parts of the world show a high prevalence of anxiety [2, 3, 38]. Anxiety should also be treated with greater concern, as it is considered an event that precedes depression and is associated with a worse quality of life, in addition to cardiometabolic diseases [2, 41].

The analysis of comparisons of the results of anxiety and depressive symptoms, with a sample divided into girls and boys with and without excess weight, represented by BMI and abdominal obesity, as well as physically active and inactive, also showed results obtained only among male groups and females, reinforcing those girls have greater symptoms of anxiety and depression. However, the influences of overweight and physical inactivity cannot be highlighted. Our results broaden the discussion about associations between overweight and depression and anxiety, as other studies demonstrate the need for higher levels due to contradictory results [42, 43]. A study using prospective data observed that neither obesity and overweight can predict anxiety, but only in males, partially observed that anxiety can predict overweight and obesity [42]. Another study also indicated that an effect can result in overweight and obesity in boys [43].

Studies with adolescents indicate that the bi-directionality of mental disorders with obesity and overweight still demonstrates the need for further investigation. These studies also doubt whether mental disorders can lead the individual to gain weight or obesity, whether obesity can trigger anxiety and depression disorders or whether such associations do not occur without this bidirectional trend [37, 44, 45]. In contrast, the study by Fox *et al*. [45], for example, investigated the association between these variables with a sample of morbidly obese adolescents and observed up to 3.5 times greater chances of developing depression and almost 5 of developing anxiety than individuals with mild obesity.

The study was carried out with a national sample of children and adolescents in Sweden [36] with greater control for factors that could interfere with the associations and positive outcomes between obesity and depression and anxiety observed that anxiety and depression cause emotional and physiological factors that can make treatment difficult. Specific genetic conditions, acquired pathologies, sedentary lifestyle, socioeconomic aspects, inadequate eating habits, traumas and psycho-emotional influences can influence body composition or interfere with human emotional vibration [36, 45]. From this perspective, there is an understanding that the relationship between anxiety, depression and obesity is transdirectional, because it involves multifactorial aspects that go beyond the aforementioned bidirectionality. Only the individual diagnosis can accurately define the true origin of the problem, which is one more way for future discussions and explanations of the results found in this study. Perhaps the heterogeneity of depression and the multifactorial profile of obesity are factors that favor the transdirectionality of this process [46].

Regarding the results of comparisons with the sample divided by sex and in groups of physically active and inactive, different contexts may explain the fact that significant results are only found between groups of girls and boys. Without a number of studies that can provide evidence for the effects of physical activity/exercise on depression and anxiety, physical activity, when performed with moderate intensity can bring greater adherence and beneficial impacts [47, 48]. A population-based study with a large sample of Canadian adolescents and with control of different varieties suggested that individuals with less than one physical activity practice per week were at greater risk of reaching higher levels of anxiety and depression, different from those who practiced physical activity at least once a week [49]. Physical activity has been studied as a psychosocial buffer against symptoms of depression and anxiety, as it can provide social support, increase self-esteem, and generate a sense of accomplishment [50]. A study of adolescents in Sweden found that regular participation in leisure-time physical activities was associated with low levels of depressive symptoms [51]. In this study, those who practiced more than 300 minutes of physical activity per week were considered physically active [52]. However, the comparisons showed that boys had a longer weekly time of physical activity, and it seems that between the sexes, this variable is important in addition to possible explanations about the differences between boys and girls about mental health [39].

Comparisons with depression and anxiety as independent variables, practice time of physical activity and overweight as dependent variables, and sex as control did not demonstrate significant results and reinforce those new investigations with different methodological designs and more robust control variables are required [34, 35]. This study, for example, presents as limitations the lack of control variables such as socioeconomic level, obesity, type and intensity of physical activity, *etc*. There was no calculation of sample size and the sample was chosen by convenience.

## CONCLUSION

The identification of a higher prevalence of girls with anxiety and depression was observed in this study and reinforced this information already demonstrated in the scientific literature. In general, the prevalence of depression was low in boys, while the prevalence of overweight, sedentary lifestyle, anxiety and depression were higher in girls. Depression, as it is considered a mental disorder with risks even of death, should continue to be investigated in both sexes. Although the variables depression, anxiety, overweight, and practice of physical activity have no associations, the multifactorial influence observed in other studies indicates that future investigations must be carried out and demonstrates that a detailed assessment must be carried out on each individual in the clinical practice of cases of anxiety and depression.

## Figures and Tables

**Fig. (1):**
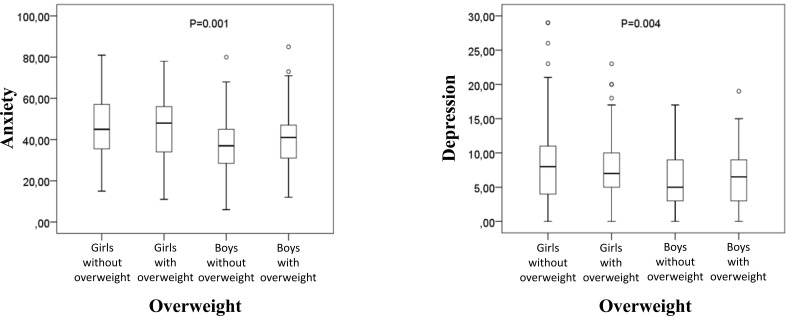
Comparison of anxiety and depression between girls and boys without and with overweight.

**Fig. (2):**
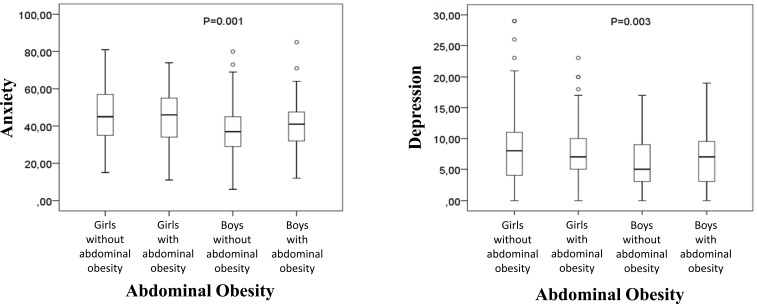
Comparison of anxiety and depression between girls and boys without and with abdominal obesity.

**Fig. (3):**
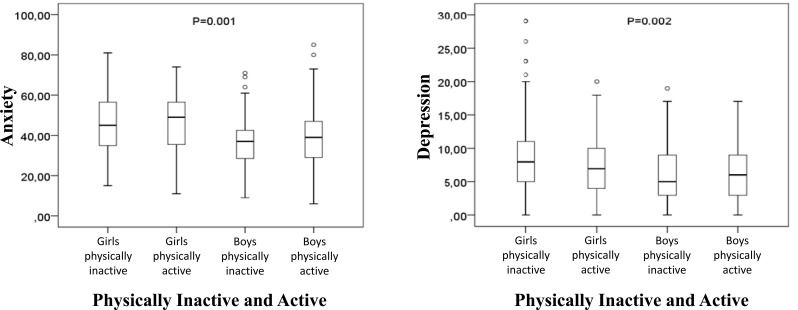
Comparisons of variables anxiety and depressive symptoms between physically inactive and physically active girls and boys.

**Table 1:** Descriptive characteristics and comparisons of anthropometry. anxiety. depression and physical activity level of Brazilian school boys and girls.

-	**Boys** **Mean±SD** **(n=145)**	**Girls** **Mean±SD** **(n=146)**	**General** **Mean±SD** **(n=291)**
**Body Mass (kg)**	58.0±14.6	56.0±15.9	56.98±15.29
**Height (cm)**	162.0±8.9*	157.3±6.8	159.67±8.27
**Waist (cm)**	71.47±10.31*	68.17±9.12	69.82±9.85
**BMI (kg/m^2^)**	21.9±5.2	22.5±5.0	22.23±5.15
**MASC (score)**	38.0 (17)	45.0 (22)*	41.0(21.0)
**CDI (score)**	5 (6)	8 (7)*	7(6)
**AF (min/week)**	325 (511.0)*	197.5 (292.5)	240.0 (365.0)
**METs/week**	1920 (3840.0)*	856 (1659.75)	1200.0(2684.0)

Note: *p<0.01; SD = standard deviation; DI = interquartile difference; BMI = body mass index; MASC = Children's Multidimensional Anxiety Scale; CDI = Children's Depression Inventory; AF = Physical Activity; METs = Metabolic equivalent of weekly physical activities.

**Table 2:** Prevalence of overweight. abdominal obesity. physical inactivity. anxiety and depression in Brazilian school children.

-	**Prevalence (95%IC)**		
-	**General (n=145)**	**Boys (n=146)**	**Girls (n=291)**
**Overweight**	30.57(25.41-36.28)	31.72(24.39-40.03)	29.44(22.34-37.64)
**Abdominal Obesity**	26.46(21.55-31.98)	24.83(18.19-32.80)	28.07(21.12-36.21)
**Physical Inactivity**	56.78(50.79-62.44)	45.52(37.40-54.01)	67.78(59.50-75.15)
**Anxiety**	19.58(15.27-24.71)	11.02(6.64-17.56)	28.08(21.12-36.21)
**Depression**	4.81 (2.76-8.11)	0.69(0.03-4.36)	8.89(5.02-15.05)

**Table 3:** Comparison with the interaction of Excess weight and minutes of physical activity in adolescents without and with anxiety and depression.

	**Without CMD (n=224)**	**Anxiety** **(n= 54)**	**Depression** **(n=12)**	**p value**
**PA (min)**	383.1±441.5	399.4±550.6	162.5±147.0	
**BMI (kg/m^2^)**	22.36±5.3	21.6±4.7	22.2±4.8	0.330
**AO (cm)**	70.2±10.2	68.2±8.8	69.1±7	0.330

Abbreviations: CMD= Common Mental Disorders; PA=Physical Activity; BMI = Body Mass Index; AO= Abdominal Obesity.

## Data Availability

The datasets are available only after requests for access are directed to project leader Geraldo Maranhão Neto as guarantor, according to the agreement shared with the participants and partners and as stated in the presentation for authorization to the ethics committee.

## LIST OF ABBREVIATIONS

WHO: World Health Organization
ACSM: American College of Sports Medicine
FME: Municipal Education Foundation
MASC: Multidimensional Anxiety Scale for Children
CDI: Children’s Depression Inventory
PAQA: Physical Activity Questionnaire for Adolescents
CDI: Children’s Depression Inventory
PAQ-A: Physical Activity Questionnaire for Adolescents
BMI: Body Mass Index
WC: Waist Circumference
MANCOVA: Multiple Covariance Analysis
